# Integrin-linked Kinase is Essential for Environmental Enrichment Enhanced Hippocampal Neurogenesis and Memory

**DOI:** 10.1038/srep11456

**Published:** 2015-06-22

**Authors:** Xu-Feng Xu, Ting Li, Dong-Dong Wang, Bing Chen, Yue Wang, Zhe-Yu Chen

**Affiliations:** 1 Department of Neurobiology, Shandong Provincial Key Laboratory of Mental Disorders, CAS Center for Excellence in Brain Science, School of Medicine, Shandong University, Jinan, Shandong 250012, People’s Republic of China

## Abstract

Environment enrichment (EE) has a variety of effects on brain structure and function. Brain-derived neurotrophic factor (BDNF) is essential for EE-induced hippocampal neurogenesis and memory enhancement. However, the intracellular pathway downstream of BDNF to modulate EE effects is poorly understood. Here we show that integrin-linked kinase (ILK) levels are elevated upon EE stimuli in a BDNF-dependent manner. Using ILK-shRNA (siILK) lentivirus, we demonstrate that knockdown of ILK impairs EE-promoted hippocampal neurogenesis and memory by increasing glycogen synthase kinase-3β (GSK3β) activity. Finally, overexpressing ILK in the hippocampus could rescue the neurogenesis and memory deficits in BDNF^+/−^ mice. These results indicate that ILK is indispensable for BDNF-mediated hippocampal neurogenesis and memory enhancement upon EE stimuli via regulating GSK3β activity. This is a new insight of the precise mechanism in EE-enhanced memory processes and ILK is a potentially important therapeutic target that merits further study.

Environmental enrichment (EE) refers to a variety of social, physical and cognitive stimuli compared to a normal environment, which has been found to have numerous effects on the brain functions^1^. EE has been reported to increase hippocampus-dependent learning and memory in various behavioral tests^2^,^3^, and have beneficial effects on brain disorders, including Alzheimer’s disease, Parkinson’s disease and various forms of brain injury^4^,^5^. The mechanism of EE functions has been shown relevant to enhance synaptogenesis, increase dendritic branching and length, as well as to promote neurogenesis in dentate gyrus (DG)^3^,^6^,^7^,^8^,^9^,^10^. The enhanced neurogenesis in DG has been reported to be involved in EE-enhanced memory performance^11^,^12^,^13^. Previous reports have demonstrated that EE could elevate brain-derived neurotrophic factor (BDNF) levels^14^, which play critical roles in hippocampal neurogenesis and memory processes^15^,^16^,^17^. Recent studies have showed that EE could not improve the hippocampal neurogenesis and memory deficits in BDNF^+/−^ mice^3^,^18^, which suggested that BDNF as a growth factor is essential in mediating the effect of EE. However, up to now the intracellular pathway downstream of BDNF to mediate EE action is still unclear.

Integrin-linked kinase (ILK) is a 59 kDa serine–threonine kinase that regulates various cellular processes, including migration, adhesion, differentiation and survival^19^,^20^. Previous studies have shown that ILK was highly expressed in a variety of brain regions including hippocampus, cerebellum and frontal cortex^21^. ILK has been shown to have critical functions in the development of the nervous system. It plays important role in neurite outgrowth and dendritic morphogenesis as well as neuronal survival in cultured neurons^21^,^22^,^23^. Mice targeted deletion of ILK in forebrain are also found to display cortical lamination and dentate gyrus defects during development in vivo^24^,^25^. In adult, knocking down of ILK in the nucleus accumbens has been reported to reverse the established locomotor cocaine sensitization^26^,^27^. However, the role of ILK in the hippocampus-dependent memory process remains unknown.

In this study, we found that EE could increase ILK levels in the hippocampus of adult mice in a BDNF-dependent manner. The upregulation of ILK was indispensable for EE-enhanced hippocampal neurogenesis and memory performance, which effect was exerted via ILK inhibited glycogen synthase kinase 3β (GSK3β) activity. Finally we demonstrated that ILK overexpression in the hippocampus could rescue the hippocampal neurogenesis and memory deficits in BDNF^+/−^ mice.

## Materials and methods

### Animals

Adult C57BL/6 J mice (Vital River Laboratories) and BDNF^+/−^ mice (2-3 months old) were housed in standard cages in a temperature controlled (22 ± 2 °C) room under diurnal condition (12 h light/dark cycle) with food and water available *ad libitum* unless noted otherwise. BDNF^+/−^ mice are described in the previous report^28^ and obtained from the JAX^®^ Mice (stock number: 002266). All animal procedures were in accordance with the guidelines of the National Institutes of Health Guide for the Care and Use of Laboratory Animals and were approved by the institutional animal care and use committee of Shandong University.

### Environmental enrichment

Eight-week-old male mice were housed in ordinary cages (non-enriched, NonE) or in enriched cages (environmental enrichment, EE; 475 × 350 × 200 mm, 15 mice per cage) equipped with running wheels, igloos, tunnels, huts, retreats and wooden toys. All mice received standard lab chow and water *ad libitum*.

### Surgery and Microinjection

Before the surgery, mice were anesthetized with 5% chloral hydrate (8 ml/kg, i.p.) and placed in the stereotaxic apparatus (8001, RWD Life Science). The coordinates (in reference to bregma) were as follows: anteroposterior (AP), −1.70 mm; lateral (L), ±1.0 mm; dorsoventral (V), −2.3 mm. The 1 × 10^9^ unit titer lentivirus with green-fluorescent protein sequence was injected into bilateral DG by the microinjection (KDS200, KD Scientific). The infusions were performed in a volume of 1 μl for 2 min and the infusion cannula was left for diffusion for an additional 3 min. The ILK shRNA sequence used for siILK lentivirus was as followed: ILK shRNA antisense, 5'ATACGGTTGAGATTCTGGC3'. The ILK plasmid used to package the ILK-overexpression lentivirus was purchased by Thermo Scientific Open Biosystems. The PGW and FUGW lentivirus vectors were used to package the target lentivirus. The GSK3β inhibitor SB216763 (Sigma, 2 mg/kg, i.p.) were given to mice every other day for 14 days based on the study before^29^.

### Contextual fear conditioning

On the training day, the mice were put into a standard fear-conditioning chamber (panlab). Each mouse was in the conditioning context for 2 min, at the end of which, three times of a 1 s, 0.5 mA shocks were given with an intertrial interval of 59 s. Following the last shock, mice remained in the chamber for 59 s before being moved back to their home cages. Twenty-four hours after training, mice were placed back to the previous conditioning chamber where training occurred and the freezing responses were recorded for 5 min without foot shock.

### Morris water maze

The Morris water maze was performed according our previous study^30^. Briefly, The apparatus included a circular water tank (120 cm diameter, 40 cm height) filled with water (22 °C) to a depth of 25 cm, and water was made opaque by using the addition of nontoxic white powder paint. A circular escape platform (6 cm in diameter) was placed 1 cm below the water surface. During the period of learning, the platform was always placed in the center of the same quadrant (target quadrant). Each trial consisted of a maximum of 60 s starting from one of the four quadrants with the mice facing the wall. If one mouse could not reach the platform in 60 s, it was guided to the platform. After reaching the platform, mice were allowed to stay there for 30 s, and then quickly dried with a towel and put under a heating lamp set at exactly 37 °C to avoid hypothermia. The mice received four trials per day in the water maze on each of the four training days. In the learning process, the escaped latencies for a single day were averaged to produce a daily mean. At day 5, the platform was removed, and mice were put to swim for 60 s. The number of platform crossing and the time spent in the four quadrants for each mouse was recorded with a video tracking system (Smart).

### Immunohistochemistry

Animals were anesthetized with 5% chloral hydrate anesthesia (8 ml/kg, i.p.) and perfused transcardially with 0.9% NaCl solution, followed by 4% paraformaldehyde (PFA), pH 7.6. Brains were post-fixed in 4% PFA overnight, followed by equilibration at 4 °C in 30% sucrose for an additional 24 h before sectioning. Brains were sliced into 40 μm coronal section series on a Microm cryostat (HM 550) at −20 °C. Immunohistological staining was performed on free-floating sections and stained in a solution containing 0.1% BSA, 0.3% Triton X-100, 10% normal goat serum, and anti-ILK mice primary antibody (Sigma, 1:200), anti-GFAP rabbit primary anibody (Millipore, 1:1000). After a series of 0.1 M phosphate buffer washes, sections were stained by using the same blocking solution as above and Alexa Fluor 488 goat anti-mice and 594 goat anti-rabbit secondary antibody (Inventrogen, 1:1000).

For BrdU detection, sections were pretreated with 50% Formamide/2 ×SSC for 1 h at 55 °C, and then rapid washed sections using 2 ×SSC. After this, sections were incubated 2 N HCl for 30 min at 37 °C and washed in 0.1 M borate buffer, pH 8.5, for 10 min. After blocking with 10% donkey serum in PBS containing 0.3% Triton X-100 for 1 h, sections were incubated with primary antibodies to BrdU (Abcam, 1:1000) and NeuN (Millipore, 1:1000) overnight at 4 °C, followed by Alexa Fluor secondary antibodies for 1 h. Briefly, to analyze cell proliferation in animals, they were injected with BrdU (Sigma, 50 mg/kg, i.p.) in 0.9% NaCl solution, and were perfused 2 h later. For analysis of survival, animals were injected with BrdU once daily for 4 days, and were perfused after 4 weeks.

### Quantification and Imaging

BrdU positive (BrdU^+^) cells and the BrdU and NeuN both positive (BrdU^+^NeuN^+^) cells within the granule cell layer of DG were pictured by the confocal fluorescence microscopy with a Carl Zeiss LSM-780 microscope fitted with a standard (1 Airy disk) pinhole and standard filter sets (Microstructural Platform of Shandong University). MetaMorph software package (Molecular Devices) with an unbiased stereological protocol as previously described was used to count^31^. The Briefly, we counted the BrdU^+^ or BrdU^+^NeuN^+^ cells in DG in every sixth section throughout the rostro-caudal extent of DG. This ensured the same cell was not counted repeatedly. The total number of BrdU^+^ or BrdU^+^NeuN^+^ cells was multiplied by six to estimate the total amount of the whole DG.

### Quantitative RT-PCR

Total RNA was isolated using TRIzol-A^+^ RNA isolation reagent (Tiangen) following the manufacturer’s protocol. A 0.5 μg aliquot of each sample was treated with DNase to avoid DNA contamination, and then was reversely transcribed using the ReverTra Ace qPCR RT Kit (catalog #FSQ-101, TOYOBO). Samples of acceptable fulfilled the following criteria: OD260/280 ≥ 1.8, OD260/230 ≥ 1.8, 28S/18S ≥ 1.5. The reaction was incubated for 15 min at 37 °C followed by 5 min at 98 °C. Quantitative real time RT-PCR was performed in a Cycler (Bio-Rad) using SYBR-Green (Roche). The primer sequences used as follows: ILK forward primer, 5'CTTCTGTGGGAACTGGTGACAC3', and reverse primer, 5'GCACAATCATGTCAAACTTGG3'; Actin forward primer: 5'CGTTGACATCCGTAAAGACCTC3' and reverse primer 5'CCACCGATCCACACAGAGTAC3'. Each sample was assayed in duplicate and the levels of mRNA were normalized for each well to the levels of beta-actin mRNA using the 2^−△△CT^ method.

### Tissue preparation and Western blot

Brains were quickly removed after decapitation at the desired time points. And then coronal sections (1 mm thick) were obtained using a mice brain slicer (Braintree Scientific). The DG regions were punched obtained at 0 °C followed by homogenization using Bullet Blender Homogenizer (Nextadvance). Rodent tissue homogenates were prepared in Tris-HCl buffer, pH 7.5, containing 150 mM NaCl, 1% NP-40, 1 mM EDTA and 1 μg/ml leupeptin, 1 μg/ml pepstatin, 3.8 μg/ml aprotinin, 1 mM PMSF, 1 mM Na_3_VO_4_ and 2 mM NaF. Extracts were clarified by centrifugation at 4 °C (14,000 *g* for 20 minutes). Supernatants were collected and eluted with SDS sample buffer, and the proteins were resolved by SDS-PAGE. The rabbit anti-ILK antibody (Cell Signaling Technology, 1:5000), the mouse anti-GSK3β antibody (BD, 1:1000), the rabbit anti-phosphor-GSK3β antibody (Cell Signaling Technology, 1:1000), the rabbit anti-BDNF (Santa Cruz, 1:1000) and the mice anti-α-Tubulin (Sigma, 1:10000) were respectively used as primary antibody. The goat anti-mouse or anti-rabbit secondary antibodies (Calbiochem, 1:1000) were used to react with the corresponding primary antibodies. Immunoreactive bands were visualized by enhanced chemiluminescence (ECL, Pierce). Densitometry analysis on the bands was calculated by Quantity One (version 4.6.2, Bio-Rad).

### Statistics

The data of contextual fear conditioning training analyzed by repeated measures two-way ANOVA. Other group differences were analyzed using two-tailed *t* test, one-way ANOVA or two-way ANOVA, which was followed by LSD *post hoc* analysis to compare means from several groups simultaneously. Significance was set at *p* < 0.05. Results are expressed as mean ± SEM. Data analyses were performed using SPSS statistical program, version 13.0.

## Result

### EE increases ILK expression in the hippocampus in a BDNF–dependent manner

**Figure 1:**
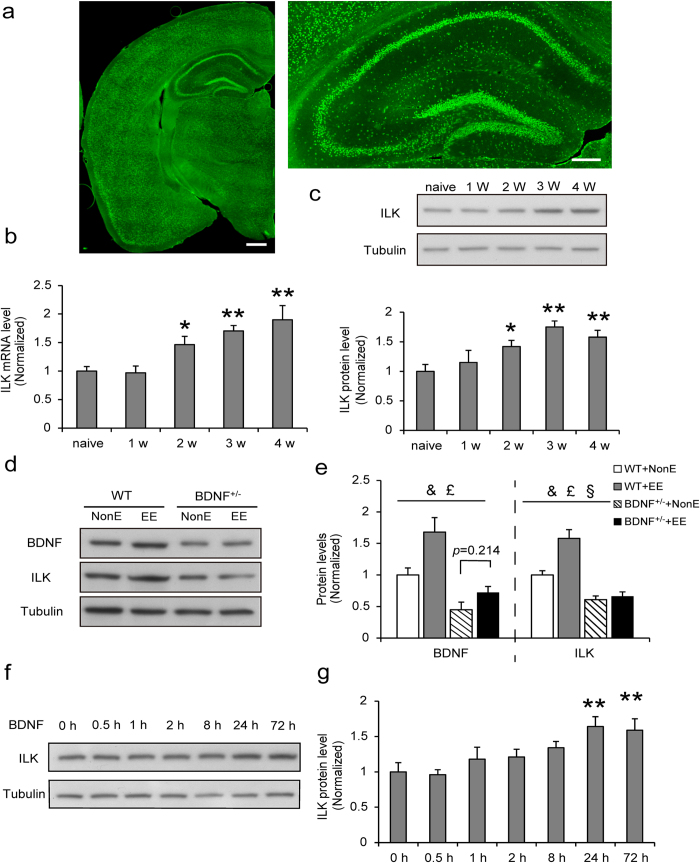
EE increases ILK expression in the hippocampus in a BDNF–dependent manner. **a**. Immunofluorescence images showing endogenous ILK protein in the adult brain (left, anteroposterior (AP), −1.70 mm; scale bar, 500 μm), and in the hippocampus (right; scale bar, 200 μm). **b, c**. relative levels of ILK mRNA (**b**) (n = 7–9 per group) and protein (**c**) (n = 4–6 per group) expression in the hippocampus of the naïve mice and the mice with EE exposure for 1, 2, 3, or 4 weeks (*p < 0.05, **p < 0.01 vs the naïve group, full-length blots are presented in Supplementary Figure 3). **d**. Representative immunoblots of ILK and BDNF proteins in the hippocampus of the WT and the BDNF^+/−^ mice, which exposed with or without EE for 4 weeks (full-length blots are presented in Supplementary Figure 3). **e**. Relative quantification ILK and BDNF protein levels (n = 7–8 per group, &: significant genotype effect; £: significant housing effect; §: significant interaction effect, WT: wild type, NonE: non-enriched). **f**. Representative immunoblots of ILK proteins in cultured hippocampal neurons with BDNF treated 0, 0.5, 1, 2, 4, 8, 24, or 72 h (full-length blots are presented in Supplementary Figure 4). **g**. Representative immunoblots of ILK protein levels (n = 3–4 per group; *p < 0.05, **p < 0.01 vs 0 h group). All values are presented as the mean ± SEM.

We used immunohistochemistry staining to examine the regions where ILK abundantly expressed in the brain and found that ILK is highly expressed in the hippocampus, a brain region critical for memories (Fig. 1a). The immunofluorescence images showed that ILK is mainly expressed in NeuN positive cells and barely found in GFAP positive cells (Supplementary figure 2), which suggests that ILK is selectively expressed in neurons. We exposed wild type mice to EE for 4 weeks and interestingly, we found that both the mRNA (Fig. 1b, F_(4,34)_ = 7.583, *p* = 0.0017, one-way ANOVA) and protein levels (Fig. 1c, F_(4,23)_ = 35.379, *p* < 0.001, one-way ANOVA) of ILK in the hippocampus were significantly elevated, which started at 2 weeks (mRNA: *p = *0.047; protein: *p* = 0.035) and sustained to 4 weeks (mRNA: *p = *0.0022; protein: *p* = 0.0029) at least. It has been reported that EE could increase BDNF expression in the hippocampus and BDNF plays an important role in EE-induced neuronal plasticity^32^. Therefore we examined whether there is relevance between EE-induced upregulation of BDNF and ILK levels in the hippocampus. We first examined whether EE exposure could change the protein levels of BDNF and ILK in the hippocampus of BDNF^+/−^ mice. To analyze the data, we defined the NonE or EE as housing factor and the WT or BDNF^+/−^ as genotype factor. Compared with WT+NonE group mice, BDNF and ILK levels were both increased in WT+EE group (Fig. 1d, e, BDNF: housing, F_(1,28)_ = 9.86; *p* = 0.004; ILK: housing, F_(1,28)_ = 43.80, *p* < 0.001; two-way ANOVA) and decreased in BDNF^+/−^ +NonE mice (Fig. 1d,e, BDNF: genotype, F_(1,28)_ = 7.58, *p* < 0.001; ILK: genotype, F_(1,28)_ = 86.51, *p* < 0.001 two-way ANOVA), suggesting that EE could promote ILK and BDNF levels and knocking down of BDNF could decrease ILK levels in the hippocampus. Furthermore, the decreased BDNF and ILK levels in BDNF^+/−^ mice showed no significant increase after 4 weeks EE exposure (Fig. 1d,e, BDNF: housing × genotype interaction, F_(1,28)_ = 1.709, *p* = 0.203; *post hoc* analysis: *p* = 0.214; ILK: housing × genotype interaction, F_(1,28)_ = 35.43, *p* < 0.001, two-way ANOVA), which suggested that BDNF knockdown abolished the upregulation of hippocampal ILK expression in response to EE exposure. To directly examine whether BDNF could regulate ILK expression or not, we then treated cultured hippocampal neurons with BDNF for various times. Quantitative immunoblot analyses revealed that ILK levels were increased after BDNF treatment in cultured hippocampal neurons in a time-dependent manner (Fig. 1f,g, F_(6,18)_ = 25.893, 24 h, *p = *0.0028; 72 h, *p = *0.0021, one-way ANOVA). These results suggest the possibility that BDNF works as an upstream signal to modulate ILK expression in EE.

### Knockdown of ILK impaired EE-enhanced hippocampus-dependent memory

**Figure 2:**
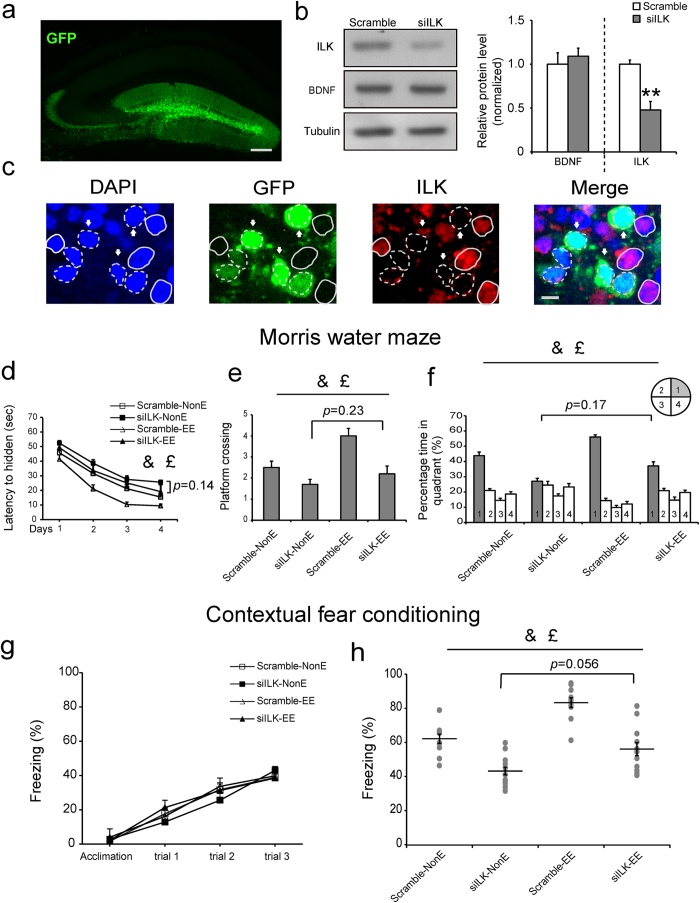
Knockdown of ILK impaired EE-enhanced hippocampus-dependent memory. **a**. The location and diffusion range of siILK lentivirus microinjected into DG (scale bar, 200 μm). **b**. Relative levels of ILK protein or BDNF protein in the DG after Scramble or siILK lentivirus injection for 4 weeks (n = 4 per group, **p < 0.01 vs the scramble group, full-length blots are presented in Supplementary Figure 4). **c**. Confocal images showed that ILK expression was rare detected in siILK lentivirus infected cells (scale bar, 10 μm). **d**–**f**. ILK knockdown impaired the spatial memory enhanced by EE in the Morris water maze test. **(d)** The escaped latency to find the hidden platform in the consecutive four days (n = 10 per group, &: significant vector effect; £: significant housing effect). **(e)** The times of platform crossing in the target quadrant in the probe test (&: significant vector effect; £: significant housing effect). **(f)** The time spent in the target quadrant in the probe test (&: significant vector effect; £: significant housing effect). **g,h**. ILK knockdown impaired the contextual fear memory enhanced by EE in the contextual fear conditioning test. **(g)** The freezing response in training process. **(h)** The freezing response 24 h after training (n = 11 per group, &: significant vector effect; £: significant housing effect). All values are presented as the mean ± SEM.

Previous studies have shown that EE could enhance hippocampus-dependent memory in various behavioral tests^33^,^34^. To examine whether the increased ILK levels in the hippocampus were functionally involved in EE-induced memory enhancement, we stereotactically injected scramble or ILK-shRNA (siILK) lentivirus, which simultaneously expressed GFP protein, into the DG of adult mice. Four weeks later, a large number of GFP-positive cells in the DG were detected in the lentivirus infected mice (Fig. 2a), suggesting that the DG cells were successfully transfected by the lentivirus. Western blotting results showed that ILK levels in DG were significantly decreased to 47.8% (*p = *0.0018, two-tailed t test) after siILK lentivirus injection compared with Scramble group, whereas BDNF levels were not affected (Fig. 2b, *p* = 0.39, two-tailed t test). Meanwhile, immunostaining results showed the rare ILK levels in siILK lentivirus infected cells (Fig. 2c). These results suggested that siILK lentivirus effectively knocked down the ILK but not BDNF levels in the hippocampus.

Next, we evaluated the effect of ILK knockdown on the EE-improved hippocampus-dependent memory. We subjected the mouse to two behavioral tests: Morris water maze and contextual fear conditioning. Mice were randomly divided into 4 groups: Scramble-NonE group, siILK-NonE group, Scramble-EE group and siILK-EE group and we defined the scramble or siILK lentivirus as vector factor. Compared with Scramble-NonE group, Scramble-EE group showed significantly decreased escape latency in the hidden platform trials, increased numbers of platform crossing and time in the target quadrant during the probe test, suggesting that EE induced the remarkable improvement of spatial memory (Fig. 2d, the fourth day: housing, F_(1,39)_ = 16.30, *p* = 0.017, 95% confidence intervals: NonE: 17.49-25.99, EE: 10.05-18.55. Figure 2e, housing, F_(1,39)_ = 11.92, *p* = 0.0014, 95% confidence intervals: NonE: 1.69-2.52, EE: 2.69–3.52. Figure 2f, housing, F_(1,39)_ = 14.61, *p = *0.001, 95% confidence intervals: NonE: 32.03–41.47, EE: 44.61–54.06, two-way ANOVA). However, siILK-NonE group exhibited the increased escape latency in the hidden platform trials, decreased the times of platform crossing and spent less time in the target quadrant during the probe test than the Scramble-NonE group, suggesting that ILK knockdown could impair the spatial memory (Fig. 2d, vector, F_(1,39)_ = 8.64, *p* = 0.006, 95% confidence intervals: Scramble: 9.41–17.91, siILK: 18.12–26.62. Figure 2e, vector, F_(1,39)_ = 20.15, *p < *0.001, 95% confidence intervals: Scramble: 2.84–3.67, siILK: 1.54–2.37. Figure 2f, vector, F_(1,39)_ = 34.19, *p* < 0.001, 95% confidence intervals: Scramble: 47.95–57.39, siILK: 28.70–38.14, two-way ANOVA). Moreover, the interaction analysis showed that there was no significant vector × housing interaction effect in the water maze test (Fig. 2d, vector × housing interaction, F_(1,39)_ = 0.122, *p* = 0.729; Fig. 2e, vector × housing interaction, F_(1,39)_ = 2.98, *p* = 0.093; Fig. 2f, vector × housing interaction, F_(1,39)_ = 2.384, *p* = 0.131, two-way ANOVA). The *post hoc* analysis exhibited that there was no statistics difference between siILK-EE and siILK-NonE group (Fig. 2d, *p = *0.14; Fig. 2e, *p = *0.23; Fig. 2f, *p = *0.17), which suggested that knockdown of ILK levels in the DG impaired EE-induced spatial memory improvement. These results indicated that EE-increased ILK levels in the hippocampus were functionally involved in EE-enhanced spatial memory.

During the test of contextual fear conditioning, mice in four groups exhibited the intact freezing response during the training process (Fig. 2g, group, F_(3,120)_ = 0.157, *p = *0.924; training trial, F_(3,120)_ = 167.855, *p < *0.001; interaction, F_(9,120)_ = 1,253, *p = *0.274, repeated measured two-way ANOVA), which suggested that hippocampal ILK knockdown and EE stimuli had no effect on contextual fear memory acquisition. We then examined the consolidation of the contextual fear memory 24 h after training, mice in Scramble-EE group showed increased freezing times when compared with that in Scramble-NonE group (Fig. 2h, housing, F_(1,43)_ = 34.40, *p* < 0.001, 95% confidence intervals: NonE: 48.12-56.83, EE: 65.99-74.69, two-way ANOVA), suggesting that EE could improve the contextual fear memory consolidation. However, mice in siILK-NonE group exhibited the significantly decreased contextual freezing responses when compared with that in Scramble-NonE group (Fig. 2h, vector, F_(1,43)_ = 59.40, *p* < 0.001, 95% confidence intervals: Scramble: 68.79-77.50, siILK: 45.32-54.02, two-way ANOVA), which suggested that knocking down ILK in the hippocampus could impair the consolidation of contextual fear memory. Moreover, the two-way ANOVA analysis showed that there was no significant vector × housing interaction effect in the contextual fear conditioning test (Fig. 2h, vector × housing interaction, F_(1,43)_ = 1.89, *p* = 0.18). The *post hoc* analysis exhibited that there was no statistics difference between siILK-EE and siILK-NonE group (Fig. 2h, *p = *0.056), suggesting that ILK knocking down blocked the EE-enhanced the contextual fear memory consolidation.

Taken together, these results indicate ILK in the hippocampus is functionally required for EE-improved hippocampus-dependent memory.

### Knockdown of ILK impaired EE-induced hippocampal neurogenesis enhancement

**Figure 3:**
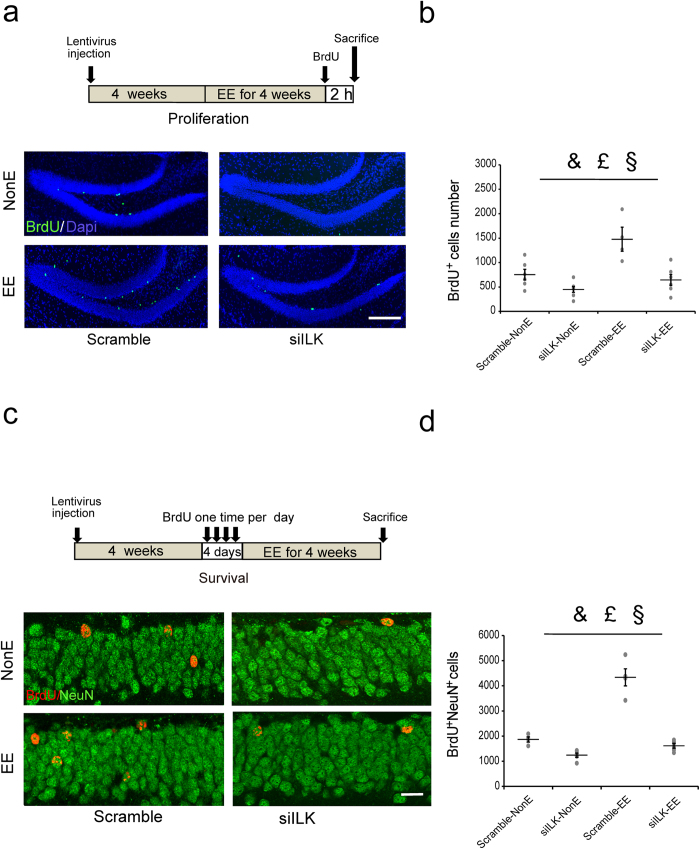
Knockdown of ILK impaired EE-induced hippocampal neurogenesis enhancement. **a,b**. Knockdown of ILK impaired the neural precursor cell proliferation promoted by EE exposure. **(a)** Representative images showed BrdU^+^ cells in DG (scale bar, 200 μm). **(b)** Quantification of BrdU^+^ cells in DG (n = 4–6 mice per group, &: significant vector effect; £: significant housing effect; §: significant interaction effect). **c**,**d**. Knockdown of ILK impaired the newborn neuron survival increased by EE exposure. **(c)** Representative images showed BrdU^+^NeuN^+^ cells in DG (scale bar, 20 μm). **(d)** Quantification of BrdU^+^ NeuN^+^ cells (n = 4–6 per group**, &**: significant vector effect; £: significant housing effect; §: significant interaction effect). All values are presented as the mean ± SEM.

The above results showed that ILK was functionally required for EE-promoted hippocampus-dependent memory; however, the underlying mechanism is still unknown. EE has been reported to increase hippocampal neurogenesis in adult mice, including the neural precursor cell proliferation and newborn neurons survival^6^,^35^, which is required for EE-induced memory improvement^12^. Next, we investigated whether ILK could participate in EE-increased neurogenesis. To this end, we examined the neural precursor cell proliferation and newborn neurons survival in the DG. Using BrdU to label newborn cells, we observed that mice in Scramble-EE group exhibited the significantly increased BrdU^+^ cells 2 h after injection of BrdU, when compared with that in Scramble-NonE group (Fig. 3a,b, housing, F_(1,21)_ = 13.58, *p* = 0.002, 95% confidence intervals: NonE: 427.00-776.33, EE: 865.97-1256.53, two-way ANOVA). These results suggested that EE could enhance progenitor proliferation in the hippocampus. However, the BrdU-labeled cells in siILK-NonE group were significantly decreased when compared with Scramble-NonE group (Fig. 3b, vector, F_(1,21)_ = 20.74, *p* < 0.001, 95% confidence intervals: Scramble: 920.14-1310.70, siILK: 372.84-722.17, two-way ANOVA), suggesting that ILK knockdown could impair the hippocampal neural precursor cell proliferation. The two-way ANOVA analysis revealed a significant vector × housing interaction effect (Fig. 3b, vector × housing interaction, F_(1,21)_ = 4.50, *p* = 0.048, two-way ANOVA), which suggested that ILK knockdown blocked the EE-promoted hippocampal neural precursor cell proliferation.

Next, we measured the survival of newborn neurons in the DG by BrdU administration once daily for four days and labeling BrdU^+^NeuN^+^ cells 28 days after last BrdU injection. After 4 weeks exposed to EE, the BrdU^+^NeuN^+^ cells in Scramble-EE group were significantly more than Scramble-NonE group (Fig. 3c,d, housing, F_(1,19)_ = 72.86, *p* < 0.001, 95% confidence intervals: NonE: 1308.41-1807.09, EE: 2728.21-3226.88, two-way ANOVA), suggesting that EE could improve the survival of newborn neurons in the hippocampus. However, mice in siILK-NonE group showed the significantly decreased BrdU^+^NeuN^+^ cells number when compared with that in Scramble-NonE group (Fig. 3d, vector, F_(1,19)_ = 101.10, *p* < 0.001, 95% confidence intervals: Scramble: 2830.74-3377.01, siILK: 1208.40-1654.43, two-way ANOVA), suggesting that knockdown of ILK could decrease the hippocampal new born neurons survival. Moreover, the interaction analysis revealed a significant vector × housing interaction effect (Fig. 3d, vector × housing interaction, F_(1,19)_ = 39.62, *p* < 0.001, two-way ANOVA), which suggested that ILK knockdown blocked the EE-promoted hippocampal newborn neurons survival. Taken together, these results indicate ILK is essential for EE increased progenitor proliferation and new born neurons survival in hippocampal neurogenesis.

### ILK participated in EE-increased neurogenesis by regulating GSK3β activity

**Figure 4:**
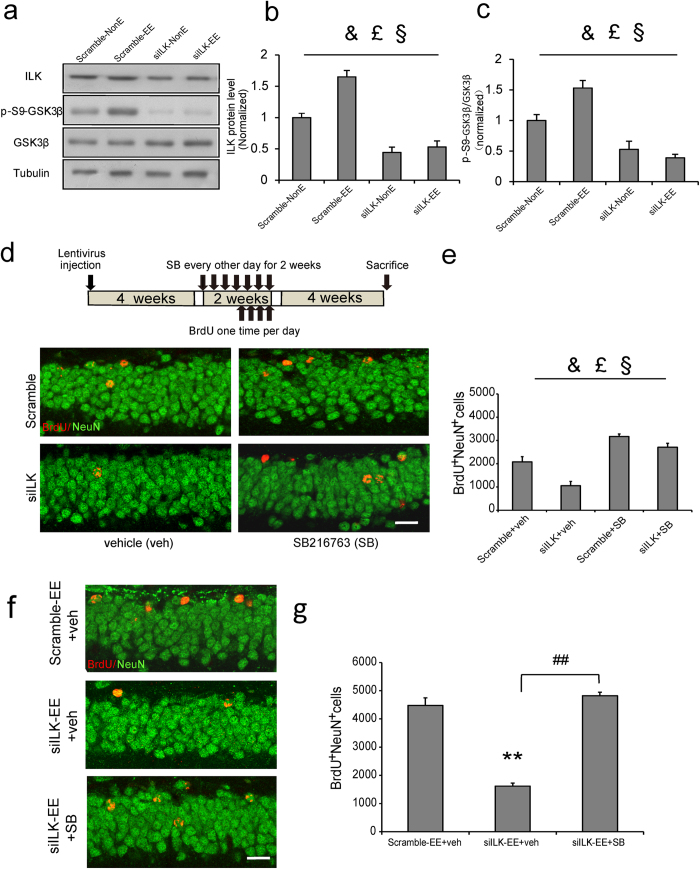
ILK participated in EE-increased neurogenesis by regulating GSK3β activity. **a–c.** ILK knockdown decreased the levels of p-s9-GSK3β whi**c**h could not be rescued by EE exposure. Representative immunoblots are shown in **a** (full-length blots are presented in Supplementary Figure 5), and the relative densitometric analysis is shown in **b,c** (n = 4–5 per group, &: significant vector effect; £: significant housing effect; §: significant interaction effect). **d**,**e.** Inhibiting GSK3β activity rescued the hippocampal n**e**wborn neuron survival impairment induced by ILK knockdown. **(d)** Representative images showed BrdU^+^NeuN^+^ cells (scale bar, 20 μm). **(e)** Quantification of BrdU^+^NeuN^+^ cells in DG (n = 5 mice p**e**r group, &: significant vector effect; £: significant treatment effect; §: significant interaction effect). **f**, **g.** Inhibiting GSK3β activity rescued the newborn neuron survival defect induced by ILK knockdown upon EE stimuli. **(f)** Representative images showed BrdU^+^NeuN^+^ cells (scale bar, 20 μm). **(g)** Quantification of BrdU^+^NeuN^+^ cells in DG (n = 4–6 mice per group, **p < 0.01 vs Scramble-EE^+^ veh group; ^##^*p* < 0.01). All values are presented as the mean ± SEM.

Although above data showed ILK was required for EE promoted neurogenesis in adult mice, it is still unclear which molecular pathway is related with ILK increased neurogenesis. Previous studies have revealed ILK could regulate GSK3β activity in cultured cells and GSK3β was shown to be involved in hippocampal neurogenesis in vivo^22^,^36^. Therefore we speculate that ILK might regulate EE-enhanced hippocampal neurogenesis through GSK3β. To confirm our hypothesis, we first examined the relationship between ILK levels and GSK3β activity in EE. Quantitative immunoblot analyses revealed that the total GSK3β protein levels did not show any significant differences in the DG of mice in four groups. However, mice in siILK-NonE group exhibited reduced GSK3β Ser-9 phosphorylation levels in the DG compared with mice in Scramble-NonE group (Fig. 4a–c, vector, F_(1,18)_ = 37.97, *p* < 0.001, two-way ANOVA), suggesting that decreased ILK levels led to elevated GSK3β activity, as the GSK3β Ser-9 phosphorylation could inhibit the GSK3β activity^37^. After four weeks of EE, ILK and GSK3β Ser-9 phosphorylation levels in Scramble-EE group were increased significantly compared with Scramble-NonE group (Fig. 4a–c, ILK: housing, F_(1,18)_ = 7.69, *p* = 0.014; p-GSK3β: housing, F_(1,18)_ = 3.33, *p* = 0.013, two-way ANOVA), which was consistent with the previous study that EE could reduce GSK3β activity^38^. However, mice in siILK-EE group showed the significantly decreased ILK and GSK3β Ser-9 phosphorylation levels when compared with those in Scramble-EE group, and showed no significant elevation when compared with mice in Scramble-NonE group (Fig. 4a–c, ILK: vector × housing interaction, F_(1,18)_ = 6.68, *p* = 0.048; p-GSK3β: vector × housing interaction, F_(1,18)_ = 7.57, *p* = 0.015, two-way ANOVA), suggesting that EE inhibited GSK3β activity were abolished by ILK knockdown. These results suggested that ILK works as an upstream signal in EE-induced GSK3β activity inhibition.

To determine whether GSK3β is functionally involved in ILK modulating hippocampal neurogenesis in adult mice, SB216763 (SB, 2 mg/kg), a specific GSK3β inhibitor^39^, was given to mice every other day for 2 weeks (4 weeks after the administration of virus, see the model of Fig. 4d) and we defined the SB or vehicle as treatment factor. Four weeks later, the vehicle treated siILK group exhibited a decreased BrdU^+^ NeuN^+^ cells number compared with the vehicle treated scramble group (Fig. 4d,e, vector, F_(1,19)_ = 33.00, *p* < 0.001, two-way ANOVA) and the SB treatment significantly increased BrdU^+^NeuN^+^ cells number in contrast to vehicle treatment in Scramble group (Fig. 4d,e, treatment, F_(1,19)_ = 112.97, *p* < 0.001, two-way ANOVA), suggesting that inhibiting GSK3β activity could enhance the hippocampal neurogenesis. Interestingly, the interaction analysis revealed a significant vector × treatment interaction effect (Fig. 4e, vector × treatment interaction, F_(1,19)_ = 4.75, *p* = 0.045, two-way ANOVA), which suggested that inhibiting GSK3β activity could rescue the ILK knockdown induced hippocampal neurogenesis deficits. These results suggest that GSK3β act downstream of ILK to regulate hippocampal neurogenesis.

Furthermore, SB treatment significantly increased the BrdU^+^NeuN^+^ cells number compared with vehicle treated mice in siILK-EE group (Fig. 4f,g, F_(2,12)_ = 80.28, *p < *0.001, one-way ANOVA), suggesting that inhibiting GSK3β activity could rescue the ILK knockdown induced hippocampal neurogenesis impairment upon EE stimuli. These results suggest ILK increase neurogenesis in EE by inhibiting GSK3β activity.

### ILK participated in EE-promoted hippocampal memory by regulating GSK3β activity in adult mice

**Figure 5:**
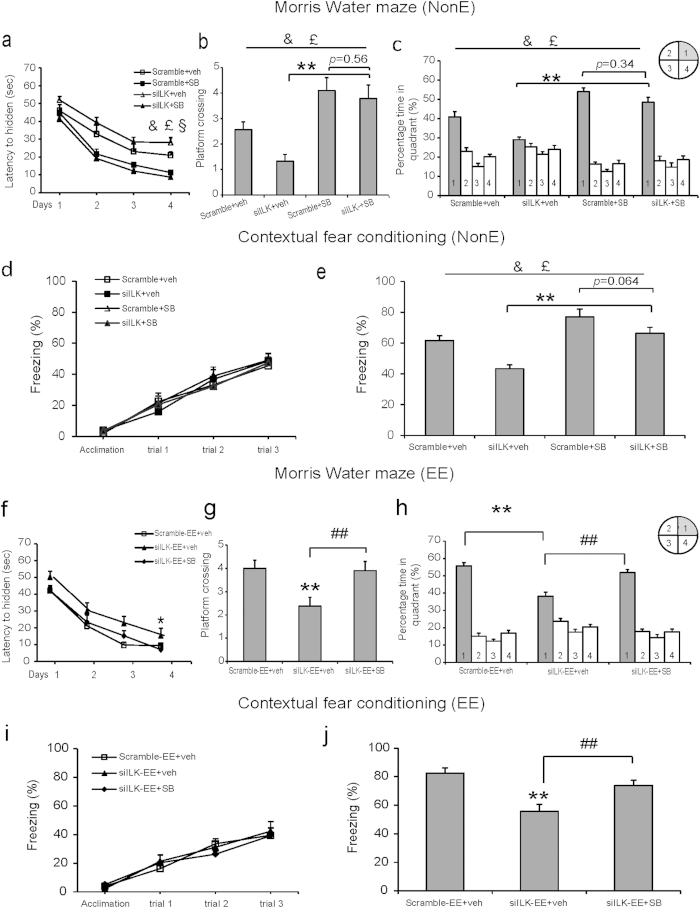
ILK participated in EE-promoted hippocampal memory by regulating GSK3β activity in adult mice. **a–c**. Inhibiting GSK3β activity rescued the spatial memory deficits induced by ILK knockdown. **(a)** Escape latency in the hidden platform trial in the consecutive four days (n = 9 per group, &: significant vector effect; £: significant treatment effect; §: significant interaction effect). **(b)** Times of platform crossing in the target quadrant in the probe test (**p < 0.01; &: significant vector effect; £: significant treatment effect) **(c)** Time spent in the target quadrant in the probe test (**p < 0.01; &: significant vector effect; £: significant treatment effect). **d,e**. Inhibiting GSK3β activity rescued the contextual fear memory defects induced by ILK knockdown. **(d)** The freezing response analysis in training process. **(e)** The freezing response 24 h after training (n = 9 per group, **p < 0.01; &: significant vector effect; £: significant treatment effect). **f–h**. Inhibiting GSK3β activity rescued the spatial memory impairment induced by ILK knockdown upon EE stimuli. **(f)** Analysis of escape latency in the hidden platform trial (n = 10 per group, *p < 0.05 vs Scramble-EE+veh group)**. (g)** Times of platform crossing in the target quadrant in the probe test (**p < 0.01 vs Scramble-EE+veh group; ^##^*p* < 0.01). **(h)** Time spent in the target quadrant in the probe test (**p < 0.01; ^##^*p* < 0.01). **i,j**. Inhibiting GSK3β activity rescued the contextual fear memory defects induced by ILK knockdown upon EE stimuli. **(i)** The freezing response analysis in training process**. (j)** The freezing response 24 h after training (n = 9 per group, **p < 0.01 vs Scramble-EE+veh group; ^##^*p* < 0.01). All values are presented as the mean ± SEM.

Our above data revealed that ILK participating in EE-increased hippocampal neurogenesis via inhibiting GSK3β activity in adult mice. Enhanced neurogenesis by inhibiting GSK3β activity has been demonstrated to promote the hippocampus-dependent memory in vivo^29^. Next we determined whether ILK-promoted hippocampal neurogenesis via GSK3β was associated with a functional behavioral consequence. Morris water maze test revealed that after given SB treatment, mice in Scramble group significantly decreased the escape latency in the hidden platform trials (Fig. 5a, the fourth day, treatment, F_(1,35)_ = 49.69, *p < *0.001, two-way ANOVA), increased number of platform crossing (Fig. 5b, treatment, F_(1,35)_ = 23.45, *p* < 0.001, two-way ANOVA) and time in the target quadrant during the probe test (Fig. 5c, treatment, F_(1,35)_ = 16.26, *p* < 0.001; two-way ANOVA), suggesting that inhibiting GSK3β activity promoted the spatial memory. The contextual fear conditioning test results showed that SB treatment had no significant effects on contextual fear memory acquisition (Fig. 5d, group, F_(3,96)_ = 0.237, *p = *0.869; training trial, F_(3,96)_ = 168.264, *p < *0.001; interaction, F_(9,96)_ = 2.977, *p = *0.005, repeated measured two-way ANOVA), but significantly increased the freezing time compared with vehicle treated mice in Scramble group during the test 24 h after training (Fig. 5e, treatment, F_(1,35)_ = 24.57, *p* < 0.001, two-way ANOVA), suggesting that inhibiting GSK3β activity improved the consolidation of contextual fear memory.

Interestingly, in the training trial, there is a significant vector × treatment interaction effect (Fig. 5a, vector × treatment interaction, F_(1,35)_ = 5.735, *p* = 0.023, two-way ANOVA). Though the two-way ANOVA analysis revealed an indistinctive significant vector × treatment interaction effect (Fig. 5b, vector × treatment interaction, F_(1,35)_ = 1.55, *p* = 0.22; Fig. 5c, vector × treatment interaction, F_(1,35)_ = 0.61, *p* = 0.44; Fig. 5e, vector × treatment interaction, F_(1,35)_ = 1.03, *p* = 0.32, two-way ANOVA), the *post hoc* analysis revealed that siILK-SB group increased the hippocampal memory compared with siILK-veh group (Fig. 5b, *p* = 0.0014; Fig. 5c, *p* = 0.0018; Fig. 5e, *p* = 0.001) and showed no significant difference with Scramble-SB group (Fig. 5b, *p* = 0.56; Fig. 5c, *p* = 0.34; Fig. 5e, *p* = 0.064), which suggested that GSK3β act downstream of ILK to regulate hippocampal-dependent memory.

Moreover, after given SB treatment, mice in siILK-EE group performed significantly better spatial memory than vehicle treatment, which was reflected by mice spending less time in hidden platform trial (Fig. 5f, the fourth day: F_(2,27)_ = 4.334, *p = *0.023, one-way ANOVA), more times of platform crossing (Fig. 5g, F_(2,27)_ = 8.01, *p = *0.002, one-way ANOVA) and more time in target quadrant in the probe test (Fig. 5h, F_(2,27)_ = 10.09, *p < *0.001, one-way ANOVA), suggesting that inhibiting GSK3β activity could rescue the ILK knockdown induced hippocampus-dependent spatial memory deficits upon EE stimuli. During the test of contextual fear conditioning, SB treated mice in siILK-EE group exhibited the significantly more freezing time than vehicle treatment (Fig. 5j, F_(2,24)_ = 22.88, *p < *0.001, one-way ANOVA), suggesting that inhibiting GSK3β activity could rescue the ILK knockdown induced hippocampus-dependent contextual fear memory impairment upon EE stimuli. These results suggest that ILK promote contextual fear memory in EE by inhibiting GSK3β activity. Taken together, these results indicate that ILK participating in EE enhanced hippocampa memory via inhibiting GSK3β activity in adult mice.

### Increasing ILK levels could rescue the impaired hippocampal neurogenesis and memory in BDNF^+/−^ mice

**Figure 6:**
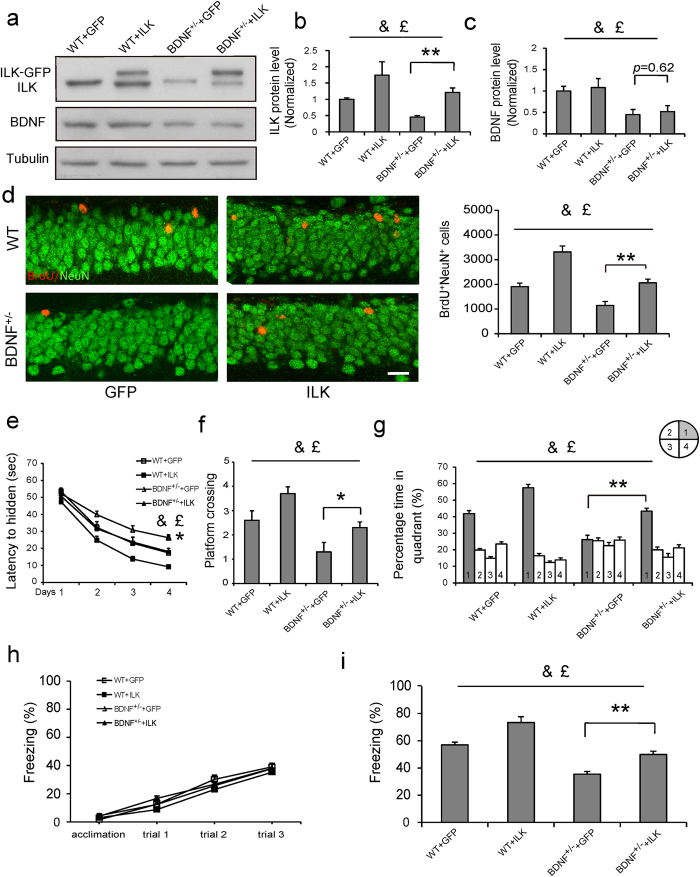
Increasing ILK levels could rescue the impaired hippocampal neurogenesis and memory in BDNF^+/−^ mice. **a–c**. ILK overexpression elevated the ILK but not BDNF levels in WT and BDNF^+/−^ mice. Representative immunoblots are shown in **a** (full-length blots are presented in Supplementary Figure 6), **a**nd the relative densitometric analysis is shown in **b,c** (n = 4–5 per group, **p < 0.01; &: significant vector effect; £: significant genotype effect). **d**. Overexpression of ILK promoted the hippocampal neurogenesis in WT and BDNF^+/−^ mice. Left: Representative images showed BrdU^+^NeuN^+^ cells (scale bar, 20 μm). Right: Quantification of BrdU^+^NeuN^+^ cells (n = 8–9 per group, **p < 0.01; &: significant vector effect; £: significant genotype effect). **e–g**. ILK overexpression promoted the spatial memory in WT and BDNF^+/−^ mice. **(e)** The escaped latency to find the platform in the consecutive four days (n = 10 per group, *p < 0.05, BDNF^+/−^ +GFP vs BDNF^+/−^ +ILK group; &: significant vector effect; £: significant genotype effect). **(f)** The tim**e**s of platform crossing in the target quadrant in the probe test (*p < 0.05; &: significant vector effect; £: significant genotype effect). **(g)** The time spent in the target quadrant in the probe test (**p < 0.01; **&**: significant vector effect; £: significant genotype effect). **h,i**. ILK overex*p*ression enhanced the contextual fear memory in WT and BDNF^+/−^ mice. **(h)** The freezing response in training process. **(i)** The freezing response 24 h after training (n = 9 per group, **p < 0.01; &: significant vector effect; £: significant genotype effect). All values are presented as the mean ± SEM.

BDNF^+/−^ mice has been reported to show hippocampal neurogenesis and memory deficits^16^,^40^, and EE exposure could not enhance hippocampus-dependent neurogenesis and memory in BDNF^+/−^ mice^3^. Our data showed that EE could increase ILK levels in the hippocampus in a BDNF-dependent manner, suggesting that ILK might work as a downstream signal of BDNF to regulate EE increased hippocampal neurogenesis and memory. Therefore, we wanted to know whether increasing ILK levels could rescue the impaired hippocampal neurogenesis and memory in BDNF^+/−^ mice. To overexpress ILK protein levels, we used lentivirus encoding ILK-GFP, a fusion protein of ILK and GFP, while the GFP encoding lentivirus was used as the control. Mice were divided into four groups, WT+GFP, WT+ILK, BDNF^+/−^ +GFP and BDNF^+/−^ +ILK. Our results revealed that injection of the ILK overexpression lentivirus significantly increased the ILK but not BDNF protein levels in WT (Fig. 6a, b, vector, F_(1,16)_ = 25.92, *p* < 0.001, two-way ANOVA) and BDNF^+/−^ mice (Fig. 6c, vector, F_(1,16)_ = 1.94, *p* = 0.187, two-way ANOVA). Then we examined whether ILK overexpression could affect the hippocampal neurogenesis in WT and BDNF^+/−^ mice. The BDNF^+/−^ mice exhibited significantly decreased BrdU^+^NeuN^+^ cells number (Fig. 6d, genotype, F_(1,32)_ = 27.68, *p* < 0.001). Compared with GFP lentivirus injection group, more BrdU^+^NeuN^+^ cells number was observed in WT+ILK group (Fig. 6d, vector, F_(1,32)_ = 42.97, *p* < 0.001, two-way ANOVA) and BDNF^+/−^ +ILK group (Fig. 6d, vector × genotype interaction, F_(1,32)_ = 2.803, *p* = 0.11, two-way ANOVA; *post hoc* analysis: *p* = 0.002), suggesting that ILK overexpression enhanced the hippocampal neurogenesis in both WT and BDNF^+/−^ mice.

We then examined whether overexpression of ILK in the hippocampus could promote the hippocampus-dependent memory in WT and BDNF^+/−^ mice using Morris water maze and contextual fear conditioning tests. Morris water maze experiment showed that BDNF^+/−^ mice exhibited a significantly impaired spatial memory in the training trial and probe test (Fig. 6e, the fourth day: genotype, F_(1,39)_ = 26.22, *p* < 0.001; Fig. 6f, genotype, F_(1,39)_ = 11.44, *p* = 0.002; Fig. 6g, genotype, F_(1,39)_ = 27.48, *p* < 0.001;). Mice with ILK overexpression significantly decreased the escape latency in the hidden platform trials (Fig. 6e, the fourth day: vector, F_(1,39)_ = 24.30, *p* < 0.001, two-way ANOVA), increased the times of platform crossing (Fig. 6f, vector, F_(1,39)_ = 18.91, *p* < 0.001, two-way ANOVA) and spent more time in the target quadrant during the probe test (Fig. 6g, vector, F_(1,39)_ = 33.28, *p* < 0.001, two-way ANOVA), suggesting that ILK overexpression enhanced the spatial memory in WT mice. The *post hoc* analysis indicated that the BDNF^+/−^ +ILK group exhibited a significant increased spatial memory compared with BDNF^+/−^ +GFP group (Fig. 6e, *p* = 0.023; Fig. 6f, *p* = 0.029; Fig. 6g, *p* = 0.0018) which suggested that ILK overexpression rescued the spatial memory deficits in BDNF^+/−^ mice.

The contextual fear conditioning test results showed the intact freezing time in 4 groups during the training process (Fig. 6h, group, F_(3,96)_ = 0.612, *p = *0.614; training trial, F_(3,96)_ = 192.698, *p < *0.001; interaction, F_(9,96)_ = 0.61, *p = *0.785, repeated measured two-way ANOVA), which suggested that hippocampal ILK overexpression and BDNF knockdown had no effect on contextual fear memory acquisition. However, when testing the long-term memory 24 h after training, the freezing time was significantly decreased in BDNF^+/−^ +GFP group and increased in WT+ILK group (Fig. 6i, genotype, F_(1,35)_ = 37.33, *p* < 0.001; vector, F_(1,35)_ = 29.45, *p* < 0.001, two-way ANOVA), suggesting that ILK overexpression promoted the contextual fear memory consolidation in WT mice. The *post hoc* analysis revealed that the BDNF^+/−^ +ILK group exhibited a significant increased freezing time compared with BDNF^+/−^ +GFP group (Fig. 6i, *p* = 0.0010), suggesting that ILK overexpression rescued the contextual fear memory deficits in BDNF^+/−^ mice. Taken together, these results indicate that ILK acts downstream of BDNF to enhance hippocampal neurogenesis and memory.

## Discussion

In this study, we observed that EE could elevate ILK protein levels in mice hippocampus in a BDNF-dependent manner. Increased ILK expression was responsible for EE-induced hippocampal neurogenesis and memory improvement by inhibiting GSK3β activity. Finally, ILK overexpression in the hippocampus could rescue the impaired hippocampal neurogenesis and memory in BDNF^+/−^ mice.

Our results provide several new insights into the mechanisms of EE-enhanced hippocampal neurogenesis and memory. First, we found ILK was involved in hippocampal neurogenesis and memory in adult mice. ILK is a serine/threonine protein kinase widely expressed in various brain regions^22^, which kinase activity is stimulated by integrins, growth factors and chemokines^41^,^42^,^43^. ILK has been shown to have indispensable functions in brain development and neurite outgrowth. Targeted deletion of ILK from embryonic mouse forebrain neuroepithelium produces transgenic mice with invasion of marginal zone, fusion of the cerebral hemispheres and scalloping of the DG^24^. To avoid the developmental neurological deficits in ILK knockout mice which could interfere with the behavior results, we locally manipulated ILK functions in the adult brain using siILK lentivirus. Our data indicated that knocking down of ILK using siILK lentivirus could impair the adult hippocampus-dependent memory and hippocampal neurogenesis, including neural precursor cell proliferation and newborn cell survival. In contrast, we also found overexpressing ILK could improve hippocampal neurogenesis and memory. These results suggest that ILK is necessary and sufficient for adult hippocampal neurogenesis and memory. Studies have shown that knockdown of ILK in nucleus accumbens blocked the induction of cocaine psychomotor sensitization and abolished cocaine-induced morphological plasticity^26^,^27^. However, the functions of ILK in hippocampal learning and memory were still unclear. Our work first convinces that ILK plays an important role in hippocampus-dependent spatial memory and the consolidation of contextual fear memory. Although previous studies showed that hippocampal neurogenesis was necessary for hippocampus-dependent memory^11^,^44^, there are still some controversies about the role of hippocampal newborn neurons in learning and memory processes^45^,^46^,^47^,^48^,^49^,^50^. Our behavioral experiments show additional evidence to support newborn neurons in the DG play important role in hippocampus-dependent memory.

Second, this is the first time demonstrating the important role of ILK in the EE-enhanced neurogenesis and memory. EE has been shown to increase adult hippocampal neurogenesis and memory in vivo. Previous studies have indicated that BDNF as a growth factor was essential for EE-increased neurogenesis and memory^3^. Some studies have shown that EE could reduce GSK3β activity and elevate AKT activity in vivo^38^,^51^. However, whether these proteins were involved in EE functions was unknown. Recent research has reported that KIF1A as an intracellular protein was found to be required for EE-increased hippocampal memory^3^. However, KIF1A was involved in EE-increased hippocampal memory by increasing synaptogenesis but not neurogenesis. Up to now, the intracellular pathway downstream of BDNF to mediate EE-increased neurogenesis and memory is still unclear. Our work indicated that EE could increase ILK levels in the hippocampus and knockdown of ILK could impair the EE-induced hippocampal neurogenesis and memory enhancement, which suggested that ILK is required for EE-promoted neurogenesis and memory.

In our study, we found that there was a significant vector and housing interaction effect on the EE-increased hippocampal neurogenesis (Fig. 3b,d), which suggested that knockdown of ILK impaired EE-induced adult neurogenesis enhancement. However, the hippocampal memory tests including Morris water maze and contextual fear conditioning did not show the significant vector and housing interaction effect, though there was no statistics difference between siILK-EE and siILK-NonE group (Fig. 2d–h), which suggested that knocking down of ILK impaired EE-induced hippocampal memory enhancement but the impairment degree was less than that of adult neurogenesis. Recent studies have shown that both EE and adult hippocampal neurogenesis was involved in hippocampal memory^11^,^44^,^48^ and EE could increase neurogenesis to enhance hippocampal memory^12^. However, EE was a complex stimuli process, which could enhance synaptogenesis, increase dendritic branching and length^3^,^7^, etc. Neurogenesis was not the sole factor EE could underlying the effect of EE on memory and EE could enhance hippocampal memory through multiple pathways.

Third, we showed ILK was involved in EE-promoted hippocampal neurogenesis and memory by inhibiting GSK3β activity. GSK3β has been shown to be involved in hippocampal neurogenesis and memory in recent studies^52^,^53^,^54^. Inhibiting GSK3β activity by SB administration could rescue the hippocampal neurogenesis and memory deficits^29^. Our results indicated that knocking down of ILK could abolish EE stimuli induced GSK3β activity inhibition, which suggested that EE inhibited GSK3β activity via ILK in the hippocampus. Moreover, inhibiting GSK3β activity by SB could rescue the ILK knockdown induced hippocampal neurogenesis and memory dificits, which suggested that ILK was involved in hippocampal neurogenesis and memory via regulating GSK3β activity. Taken together, these results indicate that ILK could inhibit GSK3β activity to contribute to EE-promoted hippocampal neurogenesis and memory. Recent studies reveal that EE inhibits the GSK3β activity in adult mice^38^,^55^,^56^, which raises a possibility that GSK3β might be involved in the molecular and behavior functions of EE. Our study provides the evidence that GSK3β is functionly involved in EE-induced hippocampal neurogenesis and memory enhancement. Although previous studies have shown that ILK could regulate GSK3β activity to affect the axonal growth and dendrite morphogenesis in vitro^22^, our results first verified that ILK could regulate GSK3β activity to participate in neurogenesis and memory processes in vivo.

Finally, our results indicated that ILK was a downstream regulator in response to BDNF-mediated neurogenesis and memory in vivo. BDNF plays important roles in neuronal signaling in various biological processes, such as gene expression, neurogenesis and synaptic plasticity^57^,^58^,^59^. Previous studies have shown that BDNF^+/−^ mice did not exhibit any increase in hippocampal neurogenesis and memory after EE^3^, which suggested that BDNF played important role in EE-induced hippocampal neurogenesis and memory enhancement. Our results have revealed that knockdown of ILK could block the EE-induced hippocampal neurogenesis and memory enhancement, suggesting both BDNF and ILK were involved in EE promoted hippocampal neurogenesis and memory. However, the relationship of BDNF and ILK in EE mediated functions was still unclear. Our data indicate that ILK levels were significantly decreased in BDNF^+/−^ mice, which could not be elevated by EE stimuli, whereas BDNF treatment could elevate ILK expression in cultured hippocampal neurons. Previous reports have shown that BDNF knockdown exhibited impaired hippocampal neurogenesis and memory in vivo^60^. Our study suggested that ILK overexpression could significantly rescue the hippocampal neurogenesis and memory deficits in BDNF^+/−^ mice, which give us the additional evidence to prove that ILK acts downstream of BDNF to enhance hippocampal neurogenesis and memory. Recent study has shown that BDNF was essential for EE-promoted neurogenesis and synaptogenesis. However, BDNF-increased KIF1A was involved in EE-enhanced hippocampus-dependent memory through synaptogenesis, but not neurogenesis^3^. So, there might be two ways for BDNF-mediated hippocampus-dependent memory enhancement upon EE stimuli, one is neurogenesis dependent and the other is synaptogenesis dependent. As we found ILK knocking down exhibited the impairment in EE-increased hippocampal neurogenesis, it is likely that EE-promoted hippocampal neurogenesis is mediated by BDNF-ILK pathway.

In our current study, we found that knockdown of BDNF-ILK pathway could impair EE-induced hippocampal neurogenesis and memory enhancement, which suggested that the BDNF-ILK pathway plays an essential role in the regulation of EE-promoted neurogenesis and memory. However, after knocking down of ILK, EE still showed a trend of enhanced memory, which suggested that ILK might not be the sole factor to mediate the functions of EE. ILK might regulate the EE functions in concert with other factors and the epigenetics could also participate in the EE process. These regulations maybe constitute to an intricate network to mediate the functions of EE. Though our results suggested that ILK was essential for EE-enhanced hippocampal neurogenesis and memory, we need more research to know the sophisticated regulation of EE from the point of systems biology.

In conclusion, to our knowledge, we determined for the first time that BDNF-dependent ILK expression participated in EE enhanced hippocampal neurogenesis and memory. We provided evidence that ILK participated in EE-increased hippocampal neurogenesis and memory by inhibiting GSK3β activity in adult mice. Finally, we found that ILK overexpression in BDNF^+/−^ mice could reverse the impaired hippocampal neurogenesis and memory. Our study will help further understanding of the precise mechanism in EE-enhanced memory processes. Considering that EE is beneficial to many brain disorders, ILK is a potentially important therapeutic target that merits further study.

## Additional Information

**How to cite this article**: Xu, X.-F. *et al.* Integrin-linked Kinase is Essential for Environmental Enrichment Enhanced Hippocampal Neurogenesis and Memory. *Sci. Rep.* **5**, 11456; doi: 10.1038/srep11456 (2015).

## Supplementary Material

Supplementary Information
