# Supplementary material for: Integrin-linked Kinase is Essential for Environmental Enrichment Enhanced Hippocampal Neurogenesis and Memory
Source: Sci Rep. 2015 Jun 22;5:11456. doi: 10.1038/srep11456 (PMC4476098; doi:10.1038/srep11456)

Supplementary information

Integrin-linked Kinase is Essential for Environmental Enrichment Enhanced Hippocampal Neurogenesis and Memory

Xu-Feng Xu, [1] **Ting Li,** [1] **Dong-Dong Wang,** [1] **Bing Chen,** [1] **Yue Wang,** [1,*] **and Zhe-Yu Chen**[1,*]

[1]Department of Neurobiology, Shandong Provincial Key Laboratory of Mental Disorders, School of Medicine, Shandong University, Jinan, Shandong 250012, People's Republic of China.

Supplementary Figure 1:

The schematic diagram for describing our experimental design.

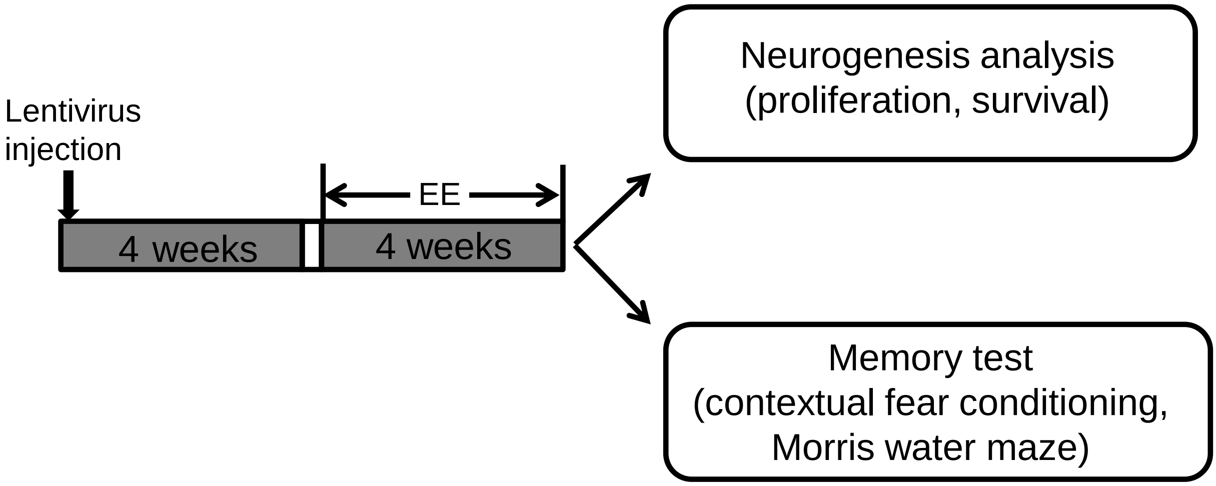

Supplementary Figure 2:

Immunofluorescence images showing endogenous ILK protein combined with NeuN and GFAP in the adult hippocampus. **A**. ILK combined with most NeuN positive cells (right). **B**. ILK hardly combined with the GFAP positive cells (left; scale bar, 20 μm).

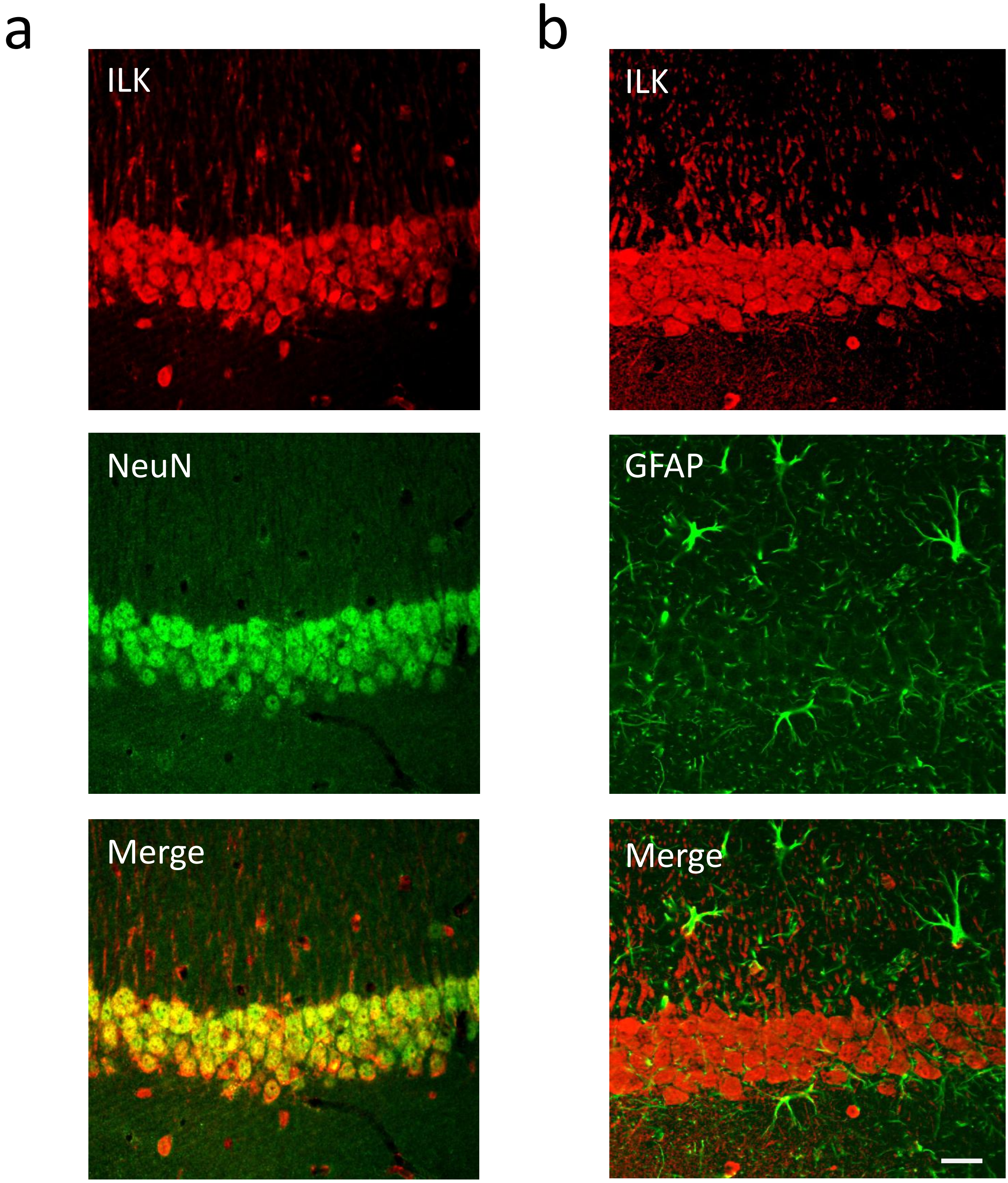

Supplementary Figure 3:

Presentation of original immunoblots.

The cropped parts of immunoblots were indicated with boxes (Figure 1)

Figure 1c

ILK

Tubulin

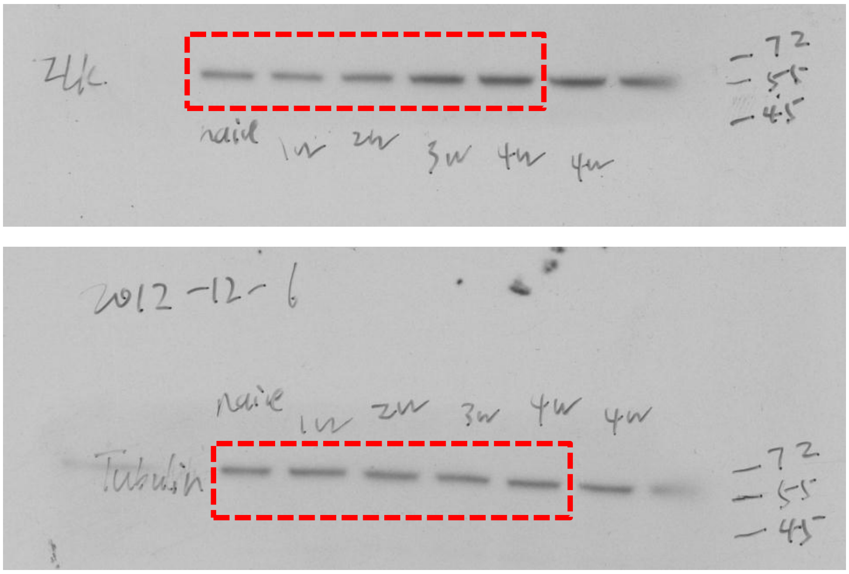

Figure 1d

BDNF

ILK

Tubulin

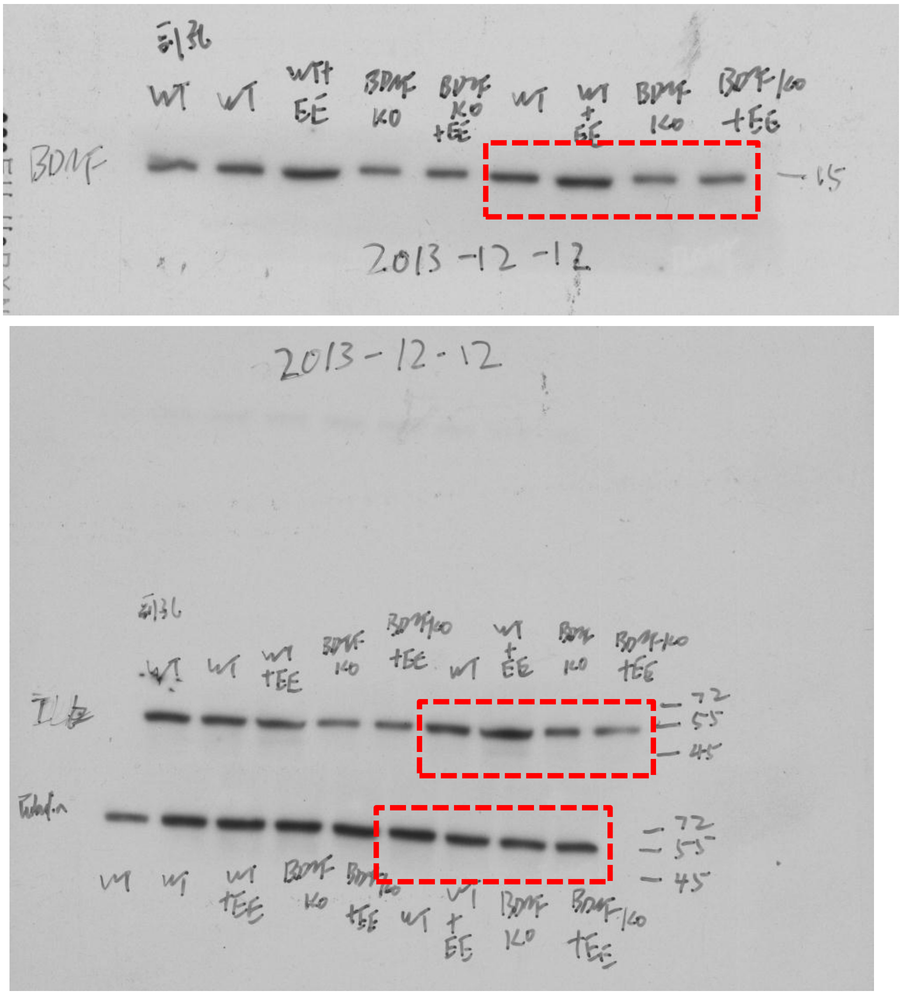

Supplementary Figure 4:

Presentation of original immunoblots.

The cropped parts of immunoblots were indicated with boxes (Figure 1 and 2)

Figure 1f

ILK

Tubulin

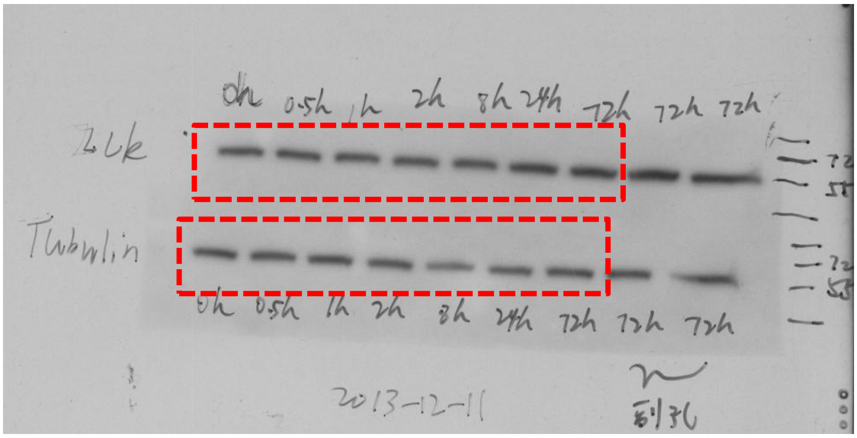

Figure 2b

ILK

BDNF

Tubulin

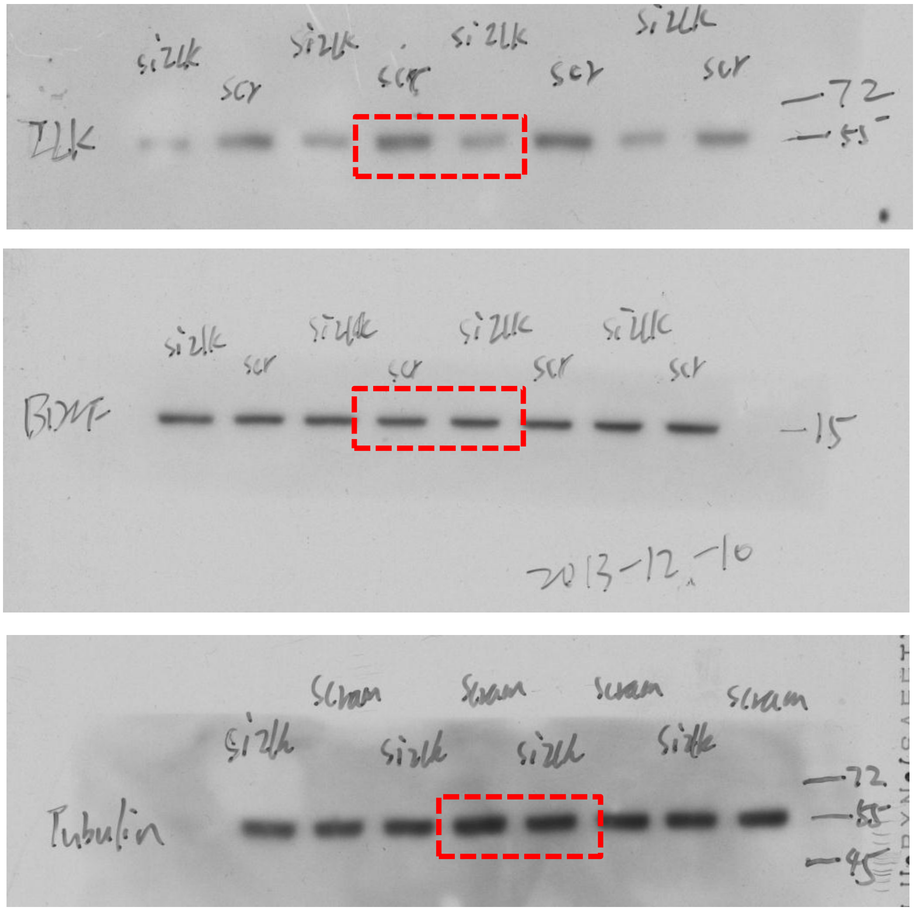

Supplementary Figure 5:

Presentation of original immunoblots.

The cropped parts of immunoblots were indicated with boxes.

(Figure 4)

Figure 4a

ILK

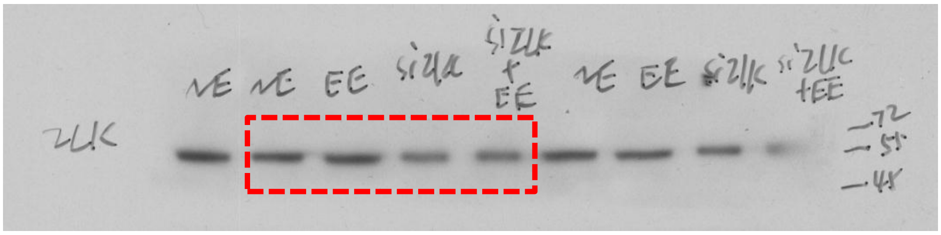

GSK3β

p-S9-GSK3β

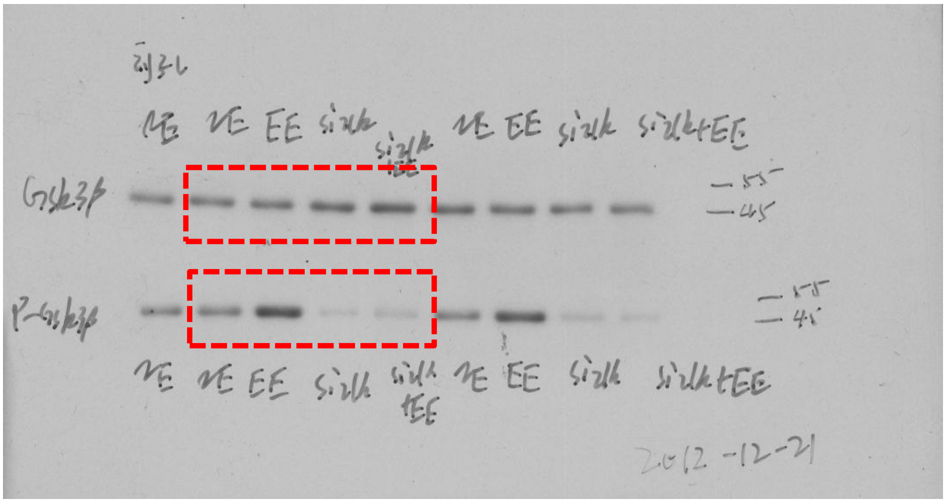

Tubulin

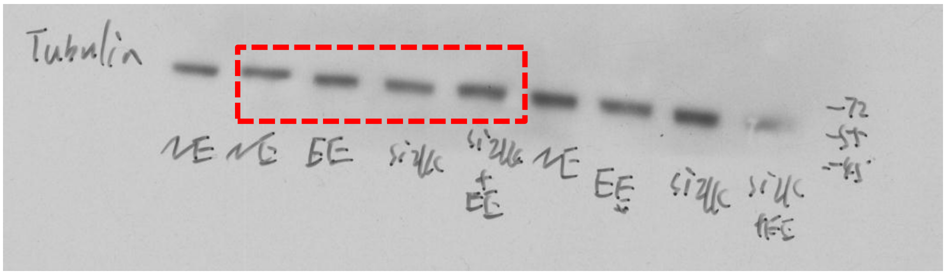

Supplementary Figure 6:

Presentation of original immunoblots.

The cropped parts of immunoblots were indicated with boxes.

(Figure 6)

Figure 6a

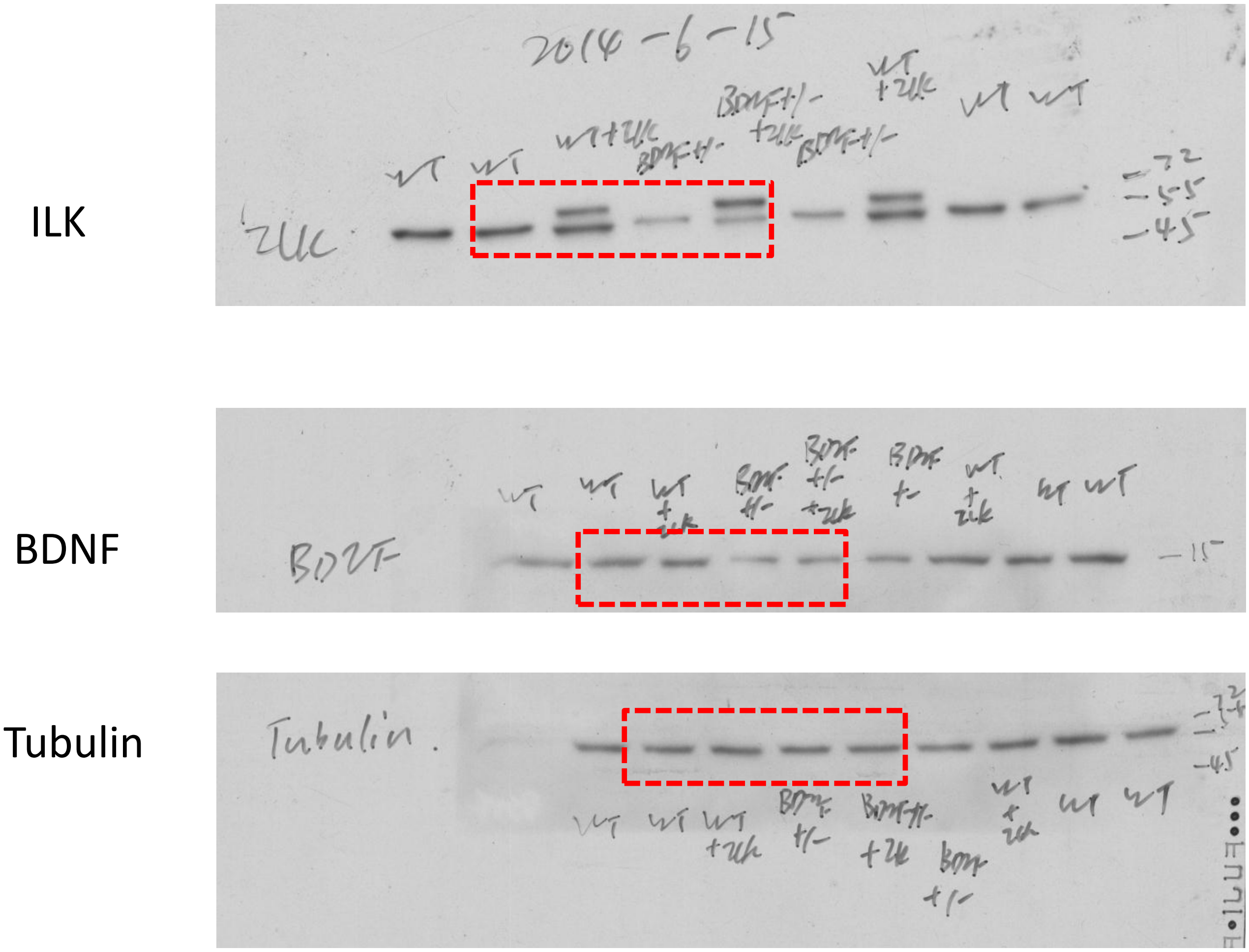

Supplement: Supplementary Information [file srep11456-s1.pdf]
